# N_0_ colorectal cancer in the elderly: prognostic role of advanced age and correlation with adjuvant chemotherapy

**DOI:** 10.1186/1471-2318-11-S1-A23

**Published:** 2011-08-24

**Authors:** M Gruppo, G Piatto, F Mazzalai, R Lorenzetti, M Di Giunta, C Terranova, A Bruttocao, C Militello

**Affiliations:** 1 Clinic of Geriatric Surgery, Hospital University of Padua, Italy; 2 Unit of Forensic Toxicology and Antidoping, Hospital University of Padua, Italy

## Background

The aim of this study is to assess the possible prognostic role of age in the risk of relapse in patients operated for N0 colorectal cancer and if this role is confirmed in a control population who underwent adjuvant chemotherapy.

## Materials and methods

129 patients who underwent radical surgery for N_0_ colorectal cancer were selected and grouped into three age classes: <65 years, between 65 and 80, >80.

A subpopulation of 44 patients with colorectal cancer in stage II was selected from the initial group for a comparison with a control population consisting of 63 patients who underwent radical surgery and adjuvant chemotherapy for neoplasms at the same stage.

## Results

In the population of 129 patients, the only significant correlation between age and clinical-pathological features is between advanced age (>80yr) and tumor location in the right colon (53.7%, p=0.04).

Risk of relapse is related both to depth of tumor invasion (42.9% in stage T4, 6.3% in stage T1, p=0.01) and advanced age (19.5% in >80yr, 4% in <65yr).

Overall survival (OS) and disease free survival (DFS) are significantly lower in patients aged over 80 than the other two classes. This significance is maintained by stratifying the 129 patients in the two age classes (<70, >70).

In the multivariate analysis age > 80yr is significantly correlated with an increased risk of relapse.

Evaluating the control group no significant correlation between relapse and clinical-pathological features was detected.

In the multivariate analysis in stage II population, advanced age doesn’t play any significant prognostic role in the risk of recurrence, while a reduction in DFS is related to depth of tumor invasion and the number of examined lymph nodes (<12).

## Conclusions

The negative prognostic role of advanced age in the risk of relapse that emerges from the first part of the study is certainly related to the increased fragility of elderly patients, which might be caused by several pathophysiological factors hypothesized by several Authors, but does not appear to be a controindication for surgery.

The comparison between only-operated patients and control ones in stage II neoplasm doesn’t show a prognostic role for advanced age in the risk of relapse.

This could be attributed to several factors, first of all the lack of subjects enrolled in the study, because of the controversial role of age in stage II candidates for adjuvant chemotherapy.

However we cannot exclude that adjuvant chemotherapy could affect the role of age as a prognostic factor.

